# Ebola Virus Entry: From Molecular Characterization to Drug Discovery

**DOI:** 10.3390/v11030274

**Published:** 2019-03-19

**Authors:** Cristiano Salata, Arianna Calistri, Gualtiero Alvisi, Michele Celestino, Cristina Parolin, Giorgio Palù

**Affiliations:** Department of Molecular Medicine, University of Padova, IT-35121 Padova, Italy; cristiano.salata@unipd.it (C.S.); arianna.calistri@unipd.it (A.C.); gualtiero.alvisi@unipd.it (G.A.); michele.celestino.uni@gmail.com (M.C.); cristina.parolin@unipd.it (C.P.)

**Keywords:** Ebola virus, *Filoviridae*, VSV, retroviral vectors, virus-like particles, pseudovirus, antivirals, small molecules, viral entry

## Abstract

Ebola Virus Disease (EVD) is one of the most lethal transmissible infections, characterized by a high fatality rate, and caused by a member of the *Filoviridae* family. The recent large outbreak of EVD in Western Africa (2013–2016) highlighted the worldwide threat represented by the disease and its impact on global public health and the economy. The development of highly needed anti-Ebola virus antivirals has been so far hampered by the shortage of tools to study their life cycle *in vitro*, allowing to screen for potential active compounds outside a biosafety level-4 (BSL-4) containment. Importantly, the development of surrogate models to study Ebola virus entry in a BSL-2 setting, such as viral pseudotypes and Ebola virus-like particles, tremendously boosted both our knowledge of the viral life cycle and the identification of promising antiviral compounds interfering with viral entry. In this context, the combination of such surrogate systems with large-scale small molecule compounds and haploid genetic screenings, as well as rational drug design and drug repurposing approaches will prove priceless in our quest for the development of a treatment for EVD.

## 1. Introduction

The genus Ebolavirus of the Filoviridae family includes five species: *Bundibugyo ebolavirus*, *Reston ebolavirus*, *Sudan ebolavirus*, *Tai Forest ebolavirus*, and *Zaire ebolavirus*. Among them, the *Zaire ebolavirus*, usually called Ebola virus (EBOV), is the main causative agent of human outbreaks, causing the Ebola virus disease (EVD) [1]. EVD is a disease of human and non-human primates that is characterized by a high fatality rate (30–90%). EBOV persists in the environment in a still unidentified animal reservoir, most likely the fruit bats, which maintains the virus in an enzootic cycle. Recently, a new ebolavirus, the Bombali virus, has been detected in free-tailed bats in Sierra Leone [2] while in China a new filovirus (Měnglà virus) was identified in rousettus bats [3] further supporting the role of bats in filovirus ecology. Occasionally, EBOV can be transmitted to non-human primates and duikers in an epizootic cycle causing outbreaks with high mortality [1]. Human infection represents a sporadic event taking place in the context of a human animal interface. Transmission is mainly due to the contact with blood or body fluids from infected humans or animals. EVD begins with nonspecific symptoms involving fever, fatigue, and muscle ache, and evolves to a severe condition associated with vomiting, diarrhea, infrequent hemorrhaging, and mental disorder leading to a comatose state and death. The convalescence phase of survivor patients lasts several months and is characterized by fatigue, joint pain as well as loss of appetite and memory. Viral RNA can be detected in specific organs, such as the testis, for more than one year after symptoms resolution [4].

Until 2014, EVD was considered a neglected disease, causing small outbreaks in remote African villages. EBOV research was focused mainly on biology aspects of viral infection or preparedness due to its potential use as bioweapon, and was limited to few laboratories equipped with biosafety level-4 (BSL-4) facilities. However, the recent large outbreak of EVD (Western Africa, 2013–2016) characterized by 28,616 cases and 11,310 deaths, highlighted the worldwide danger of this disease and its impact on global public health and economy [5].

Thus, research on the molecular dissection of EBOV life cycle received a strong stimulus and financial support with the ultimate goal of developing effective preventive and therapeutic approaches. In this review, we summarize the current knowledge of a specific step of the EBOV life cycle, the entry process, and the compounds identified so far capable of interfering with it, as well as the molecular models used to these purposes.

## 2. Ebola Virus Infection of Target Cells

**Figure 1:**
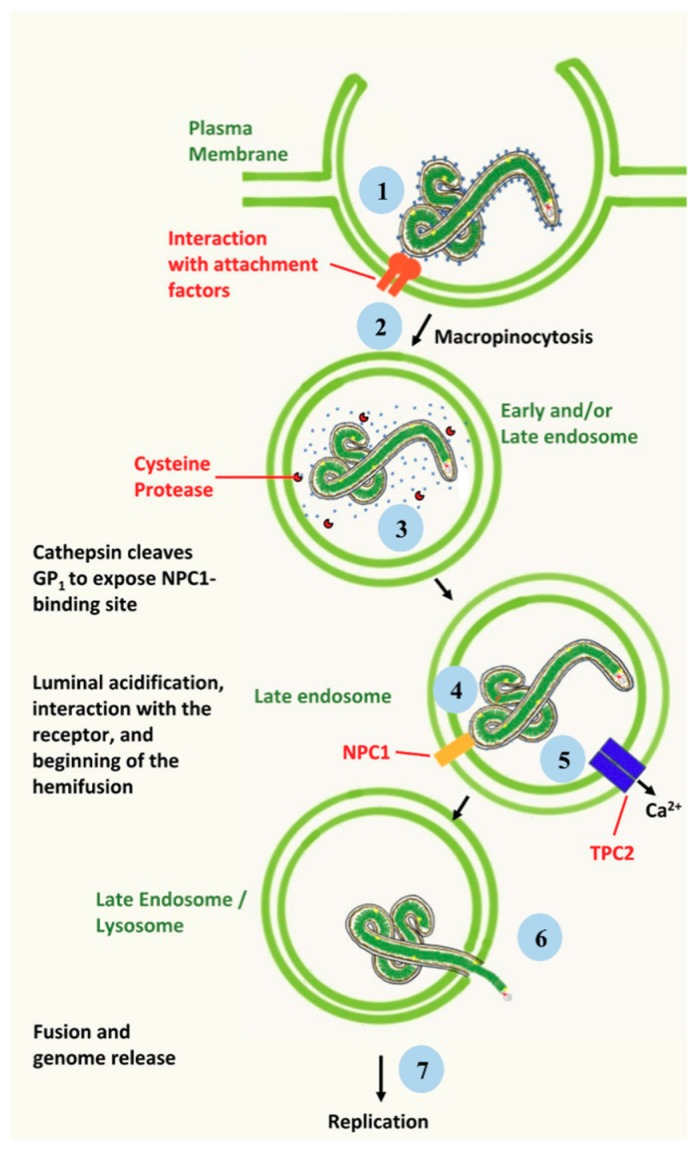
Schematic representation of EBOV entry. Following interaction with attachment factors (1), the virion is internalized by the macropinocytosis (2). Inside the membrane-bound vesicle, GP is cleaved by cysteine proteases to activate its fusogenic potential (3). Cleaved GP is then able to interact with the specific NPC1 viral receptor (4). Such event, in addition to the activity of the TPC2 calcium channel (5), helps triggering the fusion between the viral envelope and the endosomal/lysosomal membrane (6), leading to viral genome release followed by transcription and replication (7).

EBOV is an enveloped, negative-stranded RNA virus characterized by a virion of ≈80 nm of diameter and a length ranging from hundreds of nanometers to micrometers. The genome encodes for seven structural proteins: the nucleoprotein (N), the virion protein (VP) 24, VP35, VP30, VP40, the glycoprotein (GP), and the RNA-dependent RNA polymerase (L) [6]. Inside the viral particle, the ribonucleoprotein complex consists of the genomic RNA encapsidated by N, which binds to VP35, VP30 and L. The ribonucleoprotein complex interacts with the envelope, containing the GP, through the matrix protein VP40 and the minor matrix protein VP24. Viral tropism is determined by GP that allows the interaction with target cells. EBOV productively infects a broad range of cell types such as monocytes, macrophages, dendritic cells, endothelial cells, fibroblasts, hepatocytes, and adrenal cortical cells [1]. Following host cell attachment (Figure 1), the virus is internalized by macropinocytosis, a non-selective process of engulfment [7,8,9]. Binding to target cells is mediated by different attaching factors, i.e., C-type lectins, T-cell immunoglobulin and mucin domain 1, and Tyrosine kinase receptor Axl [10,11,12,13,14,15,16,17,18,19,20,21,22]. Furthermore, it has been shown that binding efficiency is related to the activity of acid Sphingomyelinase (aSMase) and to the presence of plasma membrane sphingomyelin [23]. On the viral side, EBOV attachment and entry are mediated by the surface glycoprotein GP, a class I fusion protein. In its native state, GP is a triplet of heterodimers, each composed of a receptor binding subunit (GP1) and a fusion subunit (GP2). The GP1 and GP2 subunits originate by the cleavage within the Golgi complex of a single precursor peptide, and remain associated through a disulfide bond and non-covalent interactions [17,24]. After initial internalization (Figure 1), virus particle trafficking into the endo-lysosomal pathway ends up into late endosomes, where the low-pH-dependent cysteine proteases cathepsins B and L process GP1 into a 19 kDa fusogenic form [19,23,25,26,27,28], exposing the putative receptor binding domain [29]. Subsequently, the interaction between the processed GP1 and the late endosomal/lysosomal protein Neimann-Pick C1 (NPC-1) leads to GP2-dependent fusion of the viral envelope with the endosomal limiting membrane [30,31,32]. Furthermore, it has been shown that the fusion step also requires the Two-Pore Channel 2 (TPC2) activity [33], although the specific role of TPC2 in viral entry is not entirely clear yet [34]. Finally, the viral nucleocapsid is released into the cytoplasm leading to transcription and replication of the viral genome, followed by assembly and budding of the viral progeny [35]. After the infection of primary cell targets, the viral progeny spreads to a variety of cell types and tissues, eventually resulting into a generalized organ failure [1].

## 3. Viral Models for Drug Discovery That Can Be Handled in BSL-2 Facilities: Targeting the Entry Step

Considering the high lethality of EBOV and the lack of prophylactic and therapeutic treatments, the virus can only be handled in laboratories with BSL-4 containment; thus worldwide, only few scientific institutions can conduct research and test potential countermeasures using the authentic virus. This is one of the main challenges for setting up studies focusing on the characterization of viral biology, pathogenesis and drug discovery. To overcome such issue, several alternative viral “surrogate” systems have been developed, which allowed to begin dissecting the EBOV entry pathway, and screening programs to identify entry inhibitors under BSL-2 containment. Such systems include viral pseudotypes and EBOV-like particles (eVLPs). In particular, many screening programs to identify new drugs have been performed using pseudotypes, whereas the eVLP system is considered the most reliable to molecularly dissect the EBOV life cycle.

A pseudotype is a virus or a viral vector that displays the functional envelope glycoprotein of a heterologous virus, in this case the EBOV-GP, assembled into its outer wrapping, thus acquiring a new tropism. Rhabdoviruses and retroviruses, likely due to their mechanism of budding that is independent for the presence of the envelope glycoproteins, are particularly suitable to generate pseudotypes. On the other end, eVLPs can be generated in the presence of specific EBOV proteins, and in this case, they lack a fully infectious viral genome.

Both systems have been extensively used as surrogates of the authentic virus. Such viral models can be handled under BSL-2 conditions, thus representing safe systems to identify host factors involved in viral entry as well as to identify and validate new therapeutic approaches aimed at blocking viral entry. Finally, such EBOV surrogates can be further engineered with genes encoding for reporter proteins, and used for the identification of small molecules interfering with viral entry by large scale screenings.

### 3.1. Viral Pseudotypes

#### 3.1.1. Recombinant Indiana Vesiculovirus

**Figure 2:**
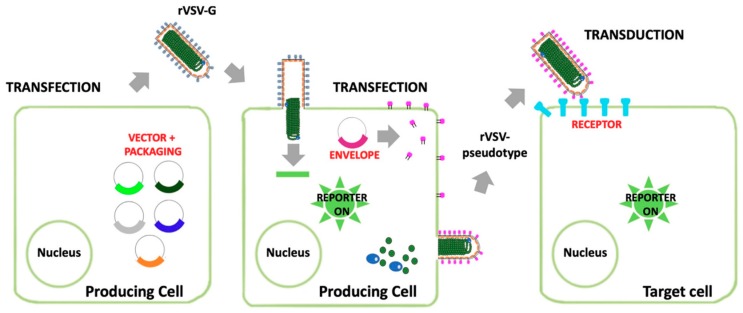
Recovery, growth and pseudotyping of rVSV-ΔG-GFP. The system is based on a plasmid encoding the viral genome, containing a reporter gene (GFP) instead of the native gene coding for the glycoprotein G, and four plasmids providing the packaging system (matrix M, polymerase L, phosphoprotein P and G). At the beginning, cells are cotransfected with the pVSV-ΔG-GFP plasmid along with the four packaging plasmids to recover the G-complemented rVSV-ΔG-GFP. To express the viral genome for the first viral rescue, a plasmid encoding the T7 RNA polymerase is also required (not shown). This virus can be used for the generation of a pseudotyped rVSV-ΔG-GFP by transducing cells preventively transfected with a plasmid encoding for the heterologous glycoproteins. Then, the pseudotyped virus can be used to transduce target cells.

The Indiana vesiculovirus, formerly named Vesicular stomatitis Indiana virus or Vesicular stomatitis virus (VSIV or VSV, and following indicated as VSV in this review) is a member of the *Rhabdoviridae* family, genus *Vesiculovirus*. VSV is a pathogenic virus for livestock while human infection is a rare event associated with an influenza-like illness. VSV can be handled in laboratory with BSL-2 containment and, therefore, it has been used as a model to study many aspects of negative-strand RNA viral entry and replication. VSV assembly occurs at the plasma membrane and is followed by the budding of virions with bullet shape of 180 nm per 75 nm from the cell surface. During budding, VSV acquires an envelope consisting of a lipid bilayer derived from the plasma membrane and spike proteins consisting of trimers of the VSV glycoprotein G (VSV-G) [36]. One of the remarkable properties of VSV is that its virions are not particularly selective with respect to the type of membrane proteins that can be incorporated into the viral envelope. Such ability coupled to that of budding in the absence of the glycoprotein G, led to the development of recombinant viruses in which the VSV-G-encoding gene was deleted (rVSV-deltaG) and replaced with a gene encoding for an unrelated envelope protein (replication competent rVSV-deltaG) [37]. A different strategy is based on the replacement of the VSV-G-encoding gene with reporter genes, such as genes encoding for fluorescent proteins (such as the green fluorescent protein -GFP- in the rVSV-deltaG-GFP), or for the luciferase [38]. Viral stocks can be generated by providing the producer cells with the envelope glycoprotein G *in trans*, for instance by means of expressing plasmids (Figure 2). When a glycoprotein or a glycoprotein complex from heterologous viruses is transiently expressed in cells transduced with such defective recombinant viruses, pseudotyped particles are efficiently released in the supernatant. Upon transduction of susceptible cells, the rVSV-deltaG pseudotypes, with a tropism dictated by the heretologous envelope glycoprotein(s), are able to complete a single round of replication, and to express the reporter gene of choice [38]. Indeed, rVSV-deltaG pseudotypes have been widely employed in studies focused on investigating mechanisms of EBOV entry into target cells, in the screening of antiviral compound libraries, for the development of tests aimed at the identification of neutralizing antibodies, and as vaccine vectors [37,38,39]. Recently, Chen and co-workers reported the development of pseudovirus infection mouse models for *in vivo* pharmacodynamics evaluation of filovirus entry inhibitors opening the possibility to easily validate data obtained by *in vitro* experiments [40].

#### 3.1.2. Retroviral Vectors (RVs)

**Figure 3:**
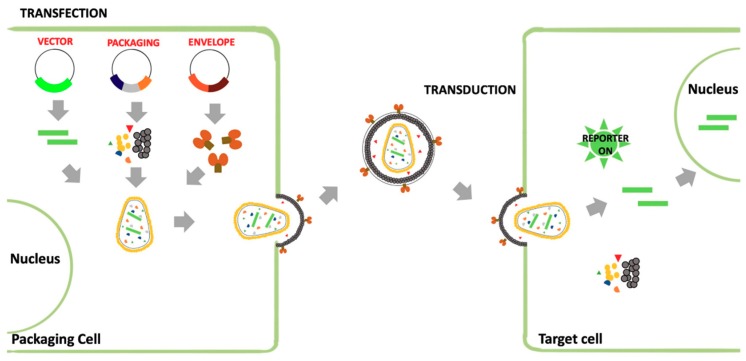
Schematic representation of the production of a pseudotyped retroviral vector. This system is based on a plasmid encoding for the retroviral vector (*cis*-acting sequences, reporter gene), and constructs expressing the packaging system factors and the heterologous envelope glycoprotein. Packaging cells are cotransfected with the different plasmids to recover pseudotyped retroviral particles in the supernatant. Pseudotyped particles can be used to transduce target cells.

Retroviruses are enveloped RNA viruses that replicate through a DNA intermediate. Indeed, upon viral entry into target cells, the viral genome is reverse transcribed into double-stranded DNA and transported to the cell nucleus, where it is permanently integrated into chromosomal DNA [41]. Viral DNA, which is known as proviral DNA, is replicated just as any other cellular gene and transferred to daughter cells. Proviral DNA is transcribed into RNA and transported to the cytoplasm, where it can be translated into structural, enzymatic and regulatory proteins. Finally, new particles will be assembled that will incorporate full length genomic RNA and bud from the cell membrane [42]. After maturation triggered by the viral protease, spherical mature viral particles of around 100 nm in diameter will be able to infect new host cells. Due to their ability to integrate their genome into the chromosomes of infected cells, retrovirus derivatives have been widely used as gene therapy vectors [43]. Lentiviruses, when compared to the other retroviruses, such as the oncogenic ones, display a more complex genome and, thus, a more complex life cycle. The etiological agent of the Acquired Immunodeficiency Syndrome, the Human Immunodeficiency Virus (HIV), is the most studied and best characterized lentivirus. Differently from gammaretroviruses, HIV can efficiently infect resting and terminally differentiated cells. This feature is one of the main reason HIV-based lentiviral vectors are currently among the most adopted vectors for gene therapy of different human diseases [44]. The last generation of HIV LVs are highly improved in terms of transgene delivery efficiency and safety. Furthermore, the backbone of RVs can be easily manipulated to express internal marker genes, in order to enable the identification of transduced cells. RVs can be easily pseudotyped with various heterologous envelopes to alter their tropism (Figure 3). While the most common example is the VSV-G pseudotyped RV, many other viral glycoproteins have also been successfully used, such EBOV and Lassa virus as the one of highly pathogenic viruses. EBOV pseudotyped RVs lead to targeted transduction of specific cell types, allowing the study of the viral entry mechanism and the screening of compound libraries with the aim of identifying compounds able to block viral entry [44].

### 3.2. Ebola Virus-Like Particles (eVLPs)

The above mentioned pseudotyped systems are however inherently flawed for the study of EBOV entry due to the morphological differences as compared to EBOV virions. Indeed, as alluded to above RV- and VSV-pseudotyped virions are either spherical or bullet shaped, respectively; remarkably different from the filamentous EBOV particles. To overcome such limitations, the EBOV-like particles (eVLPs) system was developed, which allows to generate filamentous particles, closing resembly EBOV virions. Such system relies on the peculiar properties of EBOV major matrix protein VP40, a 326 amino acid protein that is abundantly expressed during infection and plays several critical roles in the viral life cycle. In particular, VP40 is essential for assembly and budding of viral progeny by supporting the incorporation of viral ribonucleocapsids into budding virus particles. VP40 can assemble either as a hexamer, which appears to be involved in budding, or as an octamer that functions in genome replication and RNA binding [45,46,47]. When expressed alone in mammalian cells, VP40 promotes the formation of virus-like particles (eVLPs) resembling filamentous virions [48,49,50]. While VP40 alone is able to initiate budding of eVLPs, co-expressed NP and GP are incorporated into VLPs and significantly enhance their release. Only the mature forms of glycoprotein are incorporated within the eVLPs envelope, conferring to the particles the ability to infect target cells through specific EBOV receptors [51]. Thus, eVLPs have been used to study the pathway of viral entry and to identify viral entry inhibitors, in a more authentic context as compared to viral pseudotypes. Furthermore, VP40 can be easily engineered with fluorescent tags that can be exploited in imaging-based studies or with small epitope tags that facilitate its detection without modifying its budding capacity and incorporation into eVLPs [52,53,54].

One of the most suitable VLP models to study EBOV entry and its inhibition is represented by eVLP obtained by expressing VP40 fused in frame with the beta-lactamase enzyme. These VLPs allow easily detection of the fusion step during viral entry. Indeed, target cells can be incubated with a chromogenic beta-lactamase substrate that will lead to the development of a characteristic color once cleaved by the enzyme. Since the colorimetric reaction will take place immediately after the beta-lactamase is released in the cell cytosol, upon fusion of the eVLP envelope with the endosomal membrane, this system allows to distinguish between compounds blocking viral entry before and after the fusion step [54]. Therefore, VP40-beta-lactamase expressing eVLPs are precious tools for investigating the mechanism of action of active molecules.

**Figure 4:**
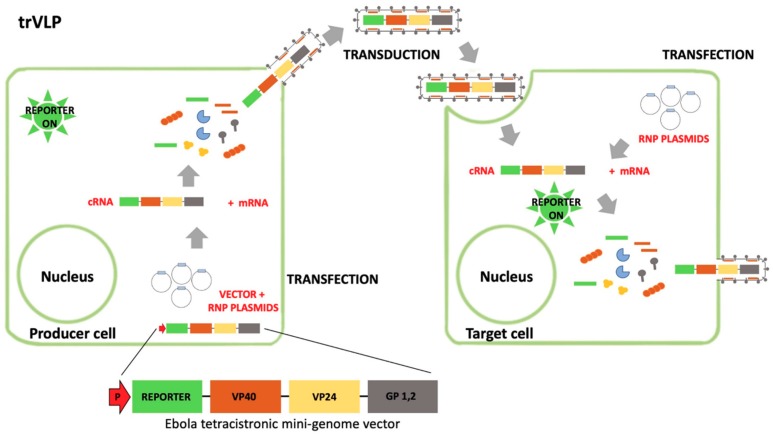
Transcription- and replication-competent eVLP (tr-eVLP). This system is based on a minigenome, encoding for a reporter gene, the viral proteins VP40, GP, and in some cases p24, co-transfected with the constructs expressing RNP proteins (N, VP35, VP30, and L). Inside the producer cells, VP40 drives the formation of eVLPs that harbor minigenome-containing nucleocapsids. These tr-eVLPs can transduce target cells and deliver the minigenome that undergoes primary transcription mediated by RNP proteins brought into the target cells within the tr-eVLPs (in the form of nucleocapsids), resulting into the expression of the reporter gene. If target cells are pre-transfected with plasmids encoding for RNPs, the minigenome is replicated and undergoes a secondary transcription (with the expression of the reporter gene) mediated by RNP proteins provided *in trans* from expression constructs. Furthermore, a new progeny of infectious tr-eVLPs is produced and can be used to transduce new target cells.

Recently, transcription- and replication-competent eVLPs (tr-eVLPs) have been developed that allow the study of almost all aspects of the viral life cycle [55]. These VLPs contain a polycistronic mini-genome that encodes for a reporter protein along with, at least, the viral proteins VP40, and GP (Figure 4). These tr-eVLPs can be continuously maintained by transferring cell culture supernatants from infected (transduced) target cells to naïve target cells [56,57]. Tr-eVLPs appear to represent the most powerful experimental system for the screening of small molecules libraries, leading to the identification of molecules that can affect different steps of the viral replication in addition to the entry one.

## 4. Ebola Virus Entry Inhibitors

The classical approach to develop an antiviral drug is based on the identification of compounds affecting the functions of specific viral proteins that play a key role in viral life cycle. On the other hand, recent approaches for the development of broad-spectrum antivirals are based on the targeting of host functions that are essential for the infection of several viruses. In both cases, any step of viral replication cycle can be targeted, such as the viral entry, genome transcription/replication, particle assembly and release. In particular, viral entry is an essential step for the establishment of the infection and thus represents an attractive target for the development of antiviral compounds. To date, many small molecules have been identified as inhibitors of EBOV entry in pre-clinical studies [58,59]. Some of these molecules are newly identified compounds, while others are already known drugs that have been shown to block EBOV infection in drug repurposing programs [58,59,60]. Among the small molecules acting as EBOV entry inhibitors that have been identified in recent years, many are cationic amphiphilic drugs (CADs), a large group of chemicals characterized by a hydrophobic aromatic ring or ring system and a hydrophilic side-chain containing an ionizable amine functional group [61]. The main antiviral activity of CADs seems to be linked to their ability to interact with different cell membranes and to accumulate in acidic intracellular compartments such as late endosomes/lysosomes that represent the gateway for EBOV entry into host cells. In addition, other small molecules with different chemical structure, or antibodies and peptides, can act as EBOV entry inhibitors affecting the virus-cell attachment, the endocytic pathway or the fusion step required by EBOV for its productive internalization inside cells [58,59,60]. In the following sections, we report the main molecules that have been identified so far as EBOV entry inhibitors. Information about the viral models used to accomplish this goal is reported in Appendix A.

### 4.1. Ion Channel Inhibitors

The antiarrhythmic drugs amiodarone, dronedarone and verapamil are ion channel blockers that have been shown to inhibit filovirus entry in cell lines and primary cells, by using a lentiviral vector pseudotyped with the EBOV or Marburg virus (MARV) glycoproteins. Interestingly, the inhibition of viral entry was effective at concentrations that are routinely reached in sera of patients treated for arrhythmia and it was confirmed by using the authentic EBOV [62,63]. Furthermore, we demonstrated that amiodarone and its main metabolite methyldiethanolamine show an additive effect improving the potential efficacy of amiodarone as an anti-EBOV compound. Time of addition experiments suggested that the entry step was targeted by amiodarone with a host-directed mechanism of action. Amiodarone seems to reduce virus binding to target cells and to slow down the progression of the viral particles along the endocytic pathway [64]. Furthermore, the drug acts by interfering with GP processing and with the fusion of the viral envelope with the endosomal membrane, blocking the virus particles inside vesicles [63]. Finally, studies with analogues of amiodarone showed that the antiviral activity is strictly correlated with the drug ability to accumulate into the endosomal compartment and to interfere with the endocytic pathway [63].

Despite these promising results *in vitro* and encouraging data from a mouse model [65], no significant clinical improvements have been reported in humans treated with amiodarone during the Western African EBOV epidemic (2013–2016) [66,67]. Furthermore, it has been recently reported that amiodarone failed to protect guinea pigs from a lethal dose of EBOV, despite the confirmation of its anti-EBOV activity in different cell types [68].

Bepridil is a calcium channel blocker that has well characterized anti-anginal properties. It has been reported that bepridil has a strong *in vitro* antiviral activity against EBOV by inhibiting a step of viral internalization before viral fusion [69]. Although bepridil may interfere with calcium-signaling required for endolysosomal fusion, it has been recently shown that it can also directly interact with the EBOV GP, by binding to a large cavity of the viral protein, thus destabilizing its prefusion conformation [70]. Interestingly, bepridil displays a significant survival benefit with a 100% survival rate for mice exposed to EBOV and treated with the drug (12 mg/kg) twice a day, beginning on the day of virus inoculation [69]. More recently, DeWald and colleague confirmed the *in vitro* antiviral activity of bepridil against MARV in Vero E6 cells and demonstrated a similar efficacy (80%–90% survival) in a murine model of MARV disease [71].

One of the latest discovered ion channel inhibitors active against the early phases of EBOV infection is tetrandrine, a compound obtained from the plant *Stephania tetrandra,* which is currently employed in the traditional Chinese medicine. Tetrandrine blocks the two pore calcium channel protein 2 (TPC2) that has been shown to be required for the release of the EBOV genome into the target cells [33]. Remarkably, tetrandrine showed therapeutic efficacy in a mouse model, with a survival rate of roughly 50% if administrated 1 day after challenge with a lethal dose of EBOV. Despite primate studies will be required before human clinical trials can begin, tetrandrine appears as a promising anti-EBOV prophylactic compound, alone or in combination with other drugs.

Finally, testing a myxobacterial natural product library, Beck and co-workers identified noricumazole A, a potassium channel inhibitor, as an new inhibitor of EBOV entry [72].

### 4.2. Antimicrobial Agents

#### 4.2.1. Antiparasitic Drugs

Chloroquine is a drug widely used in the past in the antimalarial therapy and prophylaxis before the emergence of resistant *Plasmodium* spp strains. This drug, readily available and well tolerated, is also endowed with antiviral properties, acting at two levels: the entry step and the inflammation process.

Indeed, chloroquine is a lysosomotropic agent that increases the endosomal pH affecting the normal vesicle sorting and endosome-membrane fusion. Furthermore, chloroquine displays anti-inflammatory properties by down-regulating the production of cytokines (IFN-γ and TNF-α), and the expression of TNF-α receptor [73,74]. Thus, the antiviral activity of chloroquine could be effective towards all viruses that require an acidic pH for infection of host cells, such as EBOV, and mitigate the clinical signs due to the deleterious strong immune activation following viral infection. The anti-EBOV activity of chloroquine has been reported in several *in vitro* studies adopting different viral models and cellular targets (reviewed in [61]). Despite promising evidence, *in vivo* studies did not fully support the efficacy of chloroquine for the treatment of EBOV infection. In fact, the encouraging results from two studies by Madrid and co-workers, showing a protective effect of chloroquine in mice infected with a mouse-adapted EBOV strain were not supported by more recent data, based on similar regimens, in mice, hamsters and the guinea pigs [65,75,76,77]. As well as other CADs, chloroquine may be tested for prophylactic treatment considering that it should accumulate inside host cells to display the antiviral activity.

Among drugs correlated with chloroquine, amodiaquine, hydroxychloroquine, and aminoquinoline have been shown to inhibit filovirus infection *in vitro* using a pseudotyped virus assay and the authentic EBOV [75]. Although no *in vivo* experiments have been undertaken yet, a promising result was obtained by a retrospective analysis performed on patients treated in Liberia with artesunate-amodiaquine during the Western Africa outbreak of EVD. In fact, these patients showed a lower risk of death from EVD than patients treated with artemether-lumefantrine. Although this observation lacks of several controls, the clinical effect of the artesunate-amodiaquine treatment should be better investigated as a possible therapeutic option for patients with EVD [78].

Recently, Lee and coworkers reported that the new antimalarial drug ferroquine inhibits EBOV entry, by affecting the pH dependent viral fusion step [79].

Suramin is a drug adopted to treat the trypanosome-caused African blindness. It has been demonstrated that Suramin, as a competitive inhibitor of heparin, displays antiviral activity and inhibits Chikungunya virus and EBOV infection in cellular models. However, due to its significant side effects, Suramin should be taken into consideration as therapeutic option only for highly deadly viral infections [80].

The FDA-approved compound Emetine, used for the treatment of amoebiasis, and its structural desmethyl analog have been shown to accumulate into the endosome/lysosome compartment inhibiting EBOV infection [81].

Finally, by using a VLP-based approach, the anthelmintic drugs albendazole and mebendazole have been reported to inhibit EBOV infection [82].

#### 4.2.2. Antibiotics and Antifungal Drugs

Teicoplanin, a glycopeptide antibiotic, and its derivatives potently inhibit the entry of EBOV-GP-pseudotyped viruses in various cell types [83,84]. Studies on the antiviral mechanism indicated that teicoplanin blocks EBOV entry by specifically inhibiting the activity of cathepsin L, thus avoiding the maturation of GP and the release of the viral genome into the cytoplasm [83]. The antibiotic azithromycin has been demonstrated to inhibit eVLP entry but further studies have not been performed and its mechanism of action is still largely uncharacterized [82].

Among CADs that have been proved to inhibit EBOV infection in screening experiments, there are also the antifungal drugs terconazole and triparanol, formerly used as cholesterol-lowering drugs, now withdrawn due to their numerous toxic side effects [85].

### 4.3. Psychoactive Drugs

Chlorpromazine is an anti-psychotic drug that interferes with EBOV infection [86], probably by inhibiting the internalization of virions [87].

Carette and co-workers showed that the psychoactive drug imipramine interferes with the entry of EBOV into target cells [30]. Similar effects have been reported for different psychoactive drugs, such as the antidepressant drugs sertraline, maprotiline, and trimipramine, for the anticholinergic benztropine, as well as for the anti-histamine/antiemetic compounds promethazine, diphenhylpyraline, and ketotifen [69,75,82,88]. Furthermore, two old anti-histamine drugs diphenhydramine and chlorcyclizine have been identified as potential candidates for repurposing as anti-EBOV agents. The EBOV entry inhibition is not dependent by the anti-histamine activity, but it occurs in the endosome. In fact, docking studies showed that these drugs could directly bind to the EBOV-GP [89]. Interestingly, the newer generations of anti-histamine drugs are not able to inhibit EBOV entry, suggesting that the 1st generation anti-histamines are good candidates to develop new anti-EBOV compounds by removing the unwanted histamine or muscarinic receptor interaction ability, without losing anti-filovirus efficacy [89].

Recently, a screening of a library of 1220 small molecules with predicted anti-histamine activity identified several compounds with potent inhibitory activity against EBOV infection. Data concerning the structure-activity relation will prove extremely useful to find potential scaffolds representing a favorable starting point for the rapid development of anti-EBOV therapeutic compounds [90].

### 4.4. Selective Estrogen Receptor Modulators

Drug repurposing screenings showed that several selective estrogen receptor modulators (SERMs), such as toremifene and clomiphene, are active against EBOV, inhibiting a late stage of viral entry into target cells [85,91]. Intriguingly, such activity is independent from the expression of estrogen receptors, suggesting the involvement of an alternative mode of action [91]. Although the exact inhibitory mechanism remains elusive, preliminary experimental data suggest that SERMs could interfere with the fusion of the viral envelope with the endosomal limiting membrane. Accordingly, it has been shown that toremifene directly interacts with the EBOV GP, triggering the premature release of the GP2 subunit, thus preventing the fusion process [92]. Moreover, Fan and colleagues reported that SERMs reduce the levels of cellular sphingosine and consequently an increase of calcium inside the endosomes as well as the accumulation of eVLP into TPC2^+^ endosomes. Furthermore, these compounds inhibit the ability of lentiviral vectors pseudotyped with EBOV GP to transduce target cells [93].

However, such encouraging *in vitro* results were not conclusively supported by animal studies in mice. Only one out of two studies successfully confirmed the ability of clomiphene to effectively protect mice challenged with EBOV, while in the case of toremifene, protection was obtained only in 50% of the treated animals [65,75]. Clearly, additional investigations, possibly using different animal models, are required to support the use of SERMs as anti-EBOV therapeutics.

This is particularly important since clomiphene accumulates in the eye and in the male reproductive tract, sites of EBOV persistence in patients who recovered from the infection, and therefore it could potentially act also on EBOV “reservoirs”, thus reducing the risk of viral spread [94].

### 4.5. Protein Kinase Inhibitors

Protein kinases are involved in many cellular pathways and their dysregulation is associated with diseases as cancer. Indeed, protein kinases inhibitors have already been developed and approved as anticancer drugs. Interestingly, some of them also inhibit different steps of the life cycle of several viruses, and EBOV makes no exception in this respect [82,95,96]. *In vivo* experiments demonstrated that the combination of the protein kinase inhibitors sunitinib and erlotinib can protect mice from challenges with lethal doses of EBOV [97].

An haploid genetic screening, a new approach to identify antiviral druggable targets by discovering cellular factors required for viral infection, led to the identification of the EBOV receptor NPC1 and other entry factors, such as the phosphatidylinositol-3-phospate 5-kinase (PIKfyve) [30]. Importantly, PIKfyve activity can be pharmacologically ablated by the small molecule apilimod. *In vitro* experiments showed that apilimod inhibits infections by EBOV and MARV in primary macrophages and cell lines by interfering with viral particle trafficking and blocking virions at the level of the early endosomes [98]. However, *in vivo* experiments did not support the anti-EBOV efficacy of apilimod: this PIKfyve inhibitor failed to protect EBOV-challenged mice, perhaps because of its ability to inhibit the interleukin 12 production [99].

Starting from the notion that EBOV activates the mitogen-activated protein kinase (MAPK) signaling during the entry step, Johnson and co-workers reported that pyridinyl imidazole inhibitors of p38 MAPK inhibits EBOV infection in cell lines and primary human monocyte-derived dendritic cells [100]. Indeed, pyridinyl imidazole inhibitors may represent leads for the development of effective drugs to treat EBOV infection.

Another kinase potentially druggable for anti-EBOV treatment is the Cyclin G Associated Kinase (GAK), a cellular regulator of the clathrin-associated host adaptor proteins AP-1 and AP-2. GAK regulates intracellular trafficking of multiple unrelated RNA viruses, both at the early and late stages of their life cycle, representing a potential target for broad-spectrum antivirals [101]. Recently, optimized Isothiazolo[4,3- b]pyridine-based inhibitors of GAK have been reported to efficiently inhibit the *in vitro* infection of EBOV, dengue and chikungunya viruses [102].

Finally, 1-Benzyl-3-cetyl-2-methylimidazolium iodide, an inhibitor of the eukaryotic elongation factor 2 kinase, significantly inhibits entry of single-cycle VSV harboring the EBOV GP. Interestingly, the antiviral activity of this compound is not due to its activity as kinase inhibitor but most likely to its lysosomotropic properties [103].

### 4.6. Miscellaneous Compounds That Inhibit EBOV Entry

Screening of libraries and studies on derivatives of small molecules have identified several additional compounds that can inhibit EBOV entry into target cells with different mechanisms of action.

Basu and co-workers identified a benzodiazepine derivative (also named “compound 7”), as well as compounds MBX2254 and MBX2270 as entry inhibitors of EBOV [104,105]. Several compounds with anti-EBOV entry properties were also selected after a screening analysis by Anantpadama and co-workers [106]. In addition, lead compounds can be also derived by the screening of Chinese natural herbs used in the traditional medicine [107,108].

Iminodyn 17 is an inhibitor of the GTPase activity of dynamins, a class of proteins involved in the scission of newly formed membrane vesicles, which can block EBOV entry. Nobiletin and ML9 were reported to affect the trafficking of viral particles by targeting the PI3K-Akt pathway and the myosin light chain kinase activity, respectively [79].

Retro-2 is a small molecule effective against a range of bacteria, toxins, and viruses both *in vitro* and *in vivo*. Its derivatives retro-2.1 and compound 25 have been shown to be more effective than the original molecule and to efficiently inhibit EBOV infection *in vitro* [109]. eVLPs and pseudotyped virus-based experiments indicated that these compounds block EBOV infection at the final step of viral entry [109].

At the level of late endosomes, EBOV infection can be blocked by dyphyllin derivatives that inhibit the vacuolar (H^+^)-ATPase avoiding endosome acidification [110]. The use of cysteine cathepsin inhibitors as anti-EBOV agents have also been proposed [111]. Accordingly, a recent study showed that the inhibitors of cathepsin-l, N-acetyl-l-leucyl-l-leucyl-l methional and calpeptin, block infection of a pseudotyped virus [79].

Recently, Cui and co-workers reported that diaryl-quinoline compounds are also active as entry inhibitors of EBOV [112].

The inhibition of fusion between the EBOV envelope and the endosomal membrane can be accomplished by specific peptides. The C-peptide is a synthetic peptide that corresponds to the C-terminal heptad repeat of the transmembrane subunit GP2 required for the fusion. It has been shown that the C-peptide, combined with the arginine-rich sequence of the Tat protein of HIV to improve cellular uptake, is efficiently delivered inside the endosomes where it can block EBOV infection by interfering with a membrane fusion intermediate [113]. Recently, Li and co-workers reported that Pep-3.3, a novel cyclo-peptide developed by computational approaches as able to bind to cleavage-primed EBOV GP, exhibited specific inhibitory activity against the GP-pseudotyped VSV infection [114].

U18666A is one of the typical CAD prototypes. U18666A is a cholesterol synthesis and transport inhibitor widely used in the field of lipid research and its efficacy has been tested against important human pathogens, including EBOV [30,31,61,85]. In particular, U18666A has been employed for the identification of the EBOV-intracellular receptor NPC-1 [30,31]. Although U18666A can directly interact with NPC-1, its activity seems to be due to the pleiotropic effect on the LE/Lys system [61].

## 5. Conclusions

The continuous re-emergence of EVD outbreaks in Africa with the potential risk of expansion of epidemics in other continents, as well as the possibility to use EBOV as bioweapon, makes the development of effective anti-EBOV therapeutics one of the top public health priorities. The development of BSL-2 restricted systems to study the entry of such highly pathogenic viruses outside the BSL-4 facilities simultaneously boosted both our knowledge of filovirus entry and the identification of potential, highly needed antivirals. In particular, the optimization of screening protocols in miniaturized scale allows to quickly and easily analyze large libraries of small molecules, thus providing an array of chemical structures for further modelling and structure activity relationship studies. In addition, the integration of several models allows dissecting the steps of the viral replication cycle affected by drug candidates, this shedding light on the mechanism of action of potential new antivirals. Although such viral models can speed-up the discovery of active compounds against EBOV, it is important to validate the results by using the authentic virus, in particular for compounds that directly interact with GP. In fact, the production of large amount of soluble GP forms by the original virus can interfere with the efficacy of the antiviral activity via the decoy effect played by soluble GPs. In the case of compounds acting on host cell functions, as it happens when a drug repurposing approach is undertaken, it is also possible to evaluate if multiple cellular targets contribute to the antiviral efficacy. Such information might be exploited to modify the molecules under evaluation in order to improve their antiviral activity, while attenuating their potential side effects. Considering the high pathogenicity of EBOV and the ability of viruses to develop drug resistances, the research of new molecules targeting different viral or host factors should allow obtaining efficient antiviral cocktails. In this context, evidence of synergic effect of drug combinations have been reported in *in vitro* and *in vivo* studies [97,99,115]. Finally, the intense activity of the last years on anti-filoviral research may provide benefits for other neglected infectious diseases, leading to the discovery of broad range drugs useful for the containment of outbreaks caused by other viral agents.

## Appendix A

**Table A1:** EBOV entry inhibitors and viral model systems used for their discovery.

Class &Compound	Viral Model	Validation with EBOV ^1^	Reference
**Ion channel inhibitors**			
Amiodarone	RV, VSV, VLP	YES	[62,63,64]
Bepridil	RV, VSV	YES	[69]
Dronedarone	RV	YES	[62]
Noricumazole A	RV, VSV	N/A	[72]
Tetrandrine	VSV	YES	[33]
Verapamil	RV	YES	[62]
**Antimicrobial agents**			
Albendazole	VLP	N/A	[82]
Aminoquinoline	VSV	YES	[75]
Amodiaquine	VSV	YES	[75]
Azithromycin	VLP	N/A	[82]
Chloroquine	VSV	YES	[75]
Emetine	VLP	YES	[81]
Ferroquine	VLP	N/A	[79]
Hydroxychloroquine	VSV	YES	[75]
Mebendazole	VLP	N/A	[82]
Suramin	VSV	YES	[80]
Teicoplanin	RV, VLP	N/A	[83,84]
Terconazole	RV, VSV	YES	[69,85]
Triparanol	RV, VSV	YES	[85]
**Psychoactive drugs**			
Benztropine	RV	YES	[88]
Chlorpromazine	RV	YES	[86]
Chlorcyclizine	RV	YES	[89]
Diphenhydramine	RV	YES	[89]
Diphenhylpyraline	VSV	YES	[75]
Imipramine	VSV	YES	[30]
Ketotifen	VSV	YES	[75]
Maprotiline	VLP	N/A	[82]
Promethazine	RV	YES	[88]
Sertraline	RV, VSV	YES	[69]
Trimipramine	RV	YES	[88]
**Selective estrogen receptor modulators**			
Clomiphene	VLP, VSV	YES	[69,85,91]
Toremifene	VLP	YES	[69,91]
**Protein kinase inhibitors**			
Apilimod	RV, VLP	YES	[98]
Erlotinib	VSV	YES	[97]
Pyridinyl imidazole	VLP	YES	[100]
Sunitinib	VSV	YES	[97]
1-Benzyl-3-cetyl-2-methylimidazolium iodide	VSV	N/A	[103]
**Miscellaneous compounds**			
Aloperine derivatives	RV	N/A	[107]
Benzodiazepine derivatives	RV	YES	[104]
C-peptide	VSV	YES	[113]
Calpeptin	VLP	N/A	[79]
Cyclo-peptides	VSV	N/A	[114]
Dyphyllin derivatives	VSV	YES	[110]
Ellagic acid	RV	YES	[108]
Imynodyn 17	VLP	N/A	[79]
MBX2254/2270	RV	YES	[105]
ML9	VLP	N/A	[79]
N-acetyl-l-leucyl-l-leucyl-l methional	VLP	N/A	[79]
Nobiletin	VLP	N/A	[79]
Retro 2 and derivatives	VLP, VSV	YES	[109]
U18666A	VSV	YES	[30,31]

^1^ Validation of the antiviral activity using the authentic EBOV and/or a recombinant replication competent EBOV expressing a reporter gene.
